# Regulation of Inflammation and Oxidative Stress by Formyl Peptide Receptors in Cardiovascular Disease Progression

**DOI:** 10.3390/life11030243

**Published:** 2021-03-15

**Authors:** Valentina Maria Caso, Valentina Manzo, Tiziana Pecchillo Cimmino, Valeria Conti, Pio Caso, Gabriella Esposito, Vincenzo Russo, Amelia Filippelli, Rosario Ammendola, Fabio Cattaneo

**Affiliations:** 1 Department of Medicine, Surgery and Dentistry “Scuola Medica Salernitana”, University of Salerno, 84081 Salerno, Italy; valepica8@yahoo.it (V.M.C.); vmanzo@unisa.it (V.M.); vconti@unisa.it (V.C.); afilippelli@unisa.it (A.F.); 2 Department of Molecular Medicine and Medical Biotechnology, School of Medicine, University of Naples Federico II, 80131 Naples, Italy; tiziana.pecchillocimmino@unina.it (T.P.C.); gabriella.esposito@unina.it (G.E.); rosario.ammendola@unina.it (R.A.); 3 Department of Cardiology, AORN Ospedali dei Colli-Monaldi, 80131 Naples, Italy; pio.caso@ospedalideicolli.it; 4 Department of Translational Medical Sciences, University of Campania “Luigi Vanvitelli”, 80100 Naples, Italy; vincenzo.russo@unicampania.it

**Keywords:** formyl-peptide receptors, NADPH oxidase, reactive oxygen species, annexin A1, lipoxin A4, serum-amyloid alpha, inflammation

## Abstract

G protein-coupled receptors (GPCRs) are the most important regulators of cardiac function and are commonly targeted for medical therapeutics. Formyl-Peptide Receptors (FPRs) are members of the GPCR superfamily and play an emerging role in cardiovascular pathologies. FPRs can modulate oxidative stress through nicotinamide adenine dinucleotide phosphate (NADPH) oxidase-dependent reactive oxygen species (ROS) production whose dysregulation has been observed in different cardiovascular diseases. Therefore, many studies are focused on identifying molecular mechanisms of the regulation of ROS production. FPR1, FPR2 and FPR3 belong to the FPRs family and their stimulation triggers phosphorylation of intracellular signaling molecules and nonsignaling proteins that are required for NADPH oxidase activation. Some FPR agonists trigger inflammatory processes, while other ligands activate proresolving or anti-inflammatory pathways, depending on the nature of the ligands. In general, bacterial and mitochondrial formylated peptides activate a proinflammatory cell response through FPR1, while Annexin A1 and Lipoxin A4 are anti-inflammatory FPR2 ligands. FPR2 can also trigger a proinflammatory pathway and the switch between FPR2-mediated pro- and anti-inflammatory cell responses depends on conformational changes of the receptor upon ligand binding. Here we describe the detrimental or beneficial effects of the main FPR agonists and their potential role as new therapeutic and diagnostic targets in the progression of cardiovascular diseases.

## 1. Introduction

The most important regulators of cardiac function, such as contractility, remodeling and heart rate, belong to the superfamily of G protein-coupled receptors (GPCRs) [1]. GPCRs represent the largest and the most versatile family of cell surface receptors, and since they are involved in a wide variety of physio-pathological processes they are commonly targeted for medical therapies [2]. Formyl-Peptide Receptors (FPRs) belong to the sensitive pertussis toxin-GPCR superfamily and play an emerging role in cardiovascular pathologies [3,4,5,6,7], neuronal diseases [8,9] and cancer progression [10,11,12]. FPRs family consist of three members (FPR1, FPR2 and FPR3) widely expressed in several tissues and cell types, where they modulate several biological functions, such as angiogenesis, cell proliferation and protection against cell death [9].

Initially discovered as the receptor for the formylated bacterial product N-formyl-methionine-leucyl-phenylalanine (N-fMLF), FPR1 is highly expressed in cells of bone marrow and immune system, but also in lungs, brain, and gastrointestinal tract cells [13,14]. FPR2 is the most promiscuous and widespread receptor in the FPR family. It is mainly expressed in cells of the bone marrow, immune system, gastrointestinal tract, female organ tissues, endocrine glands, brain, liver, gallbladder, and pancreas [13,14]. FPR2 is also functionally expressed on the nuclear membrane since it shows a nuclear localization sequence in the third cytoplasmic loop [15].

The biological role of FPR3 is not completely known, which is mainly expressed in monocytes and dendritic cells but not in neutrophils. It is not localized on the cell surface, like its counterpart receptors, but it resides in intracellular vesicles [13]. Most FPR ligands induce cell chemotaxis, calcium flux and phagocytosis. Some FPR agonists elicit inflammatory processes while other ligands activate proresolving or anti-inflammatory pathways, depending on the nature of the ligands. In general, bacterial and mitochondrial formylated peptides activate a proinflammatory cell response, while Annexin A1 (ANXA1) and Lipoxin A4 (LXA4) are anti-inflammatory FPR2 ligands [16]. The switch between FPR2-mediated pro- and anti-inflammatory cell responses depends on conformational changes of the receptor due to ligand binding; anti-inflammatory ligands such as ANXA1 induce the formation of FPR homodimers and, in turn, the release of inflammation-resolving cytokines. On the other hand, inflammatory ligands such as serum-amyloid alpha (SAA) do not cause receptor homodimerization [12,17].

ANXA1 is a 37-kDa member of a superfamily of 13 annexin proteins widely expressed in cells of myeloid origin [18]. The proresolving effects of this protein are mediated by the L-selectin shedding resulting in reduced neutrophil adhesion to the endothelium and limited transmigration [18].

Therefore, FPRs can be stimulated by a plethora of endogenous or exogenous ligands, with different chemical properties [12,19], suggesting that they can be considered pattern recognition receptors able to recognize pathogen-associated molecular patterns (PAMPs) and damage-associated molecular pattern (DAMPs) [20,21]. Both PAMPs and DAMPs cause a systemic inflammatory response syndrome by activating innate immunity [12]. DAMPs include proteins, nucleic acids, extracellular matrix components, and lipid mediators released from necrotic cells to guide neutrophils to sites of sterile inflammation. DAMPs take part in the early reaction after stroke onset and contribute to the acute conditions that result from sterile inflammation, also in ischemia-reperfusion injury (IRI) [22]. Among DAMPs, mitochondrial formylated peptides released from necrotic or damaged cells can interact with members of the FPR family triggering neutrophil-mediated organ injury [21]. Furthermore, mitochondrial dysfunctions contribute to sterile inflammation, by releasing mitochondrial N-formyl peptides that can bind FPRs [23], in a variety of cardiac diseases including coronary artery disease, left ventricular hypertrophy and heart failure [24,25,26].

An increased risk of cardiac injury correlates with systemic inflammation such as in chronic obstructive pulmonary disease (COPD)-associated systemic inflammation [27,28]. Indeed, hypoxemia observed in patients affected by COPD can lead to pulmonary vasoconstriction and vascular remodeling, resulting in right-ventricular diastolic dysfunction [29]. Moreover, hypoxemia in patients with COPD can be also related to an impaired cardiac repolarization by increasing the risk of ventricular arrhythmias and sudden cardiac death [29,30]. FPRs contribute to the COPD progression. FPR1 is involved in impaired epithelial repair responses associated with cigarette smoke, which is considered one of the leading causes of COPD [31]. Moreover, decreased FPR2 and FPR3, as well as defective ANXA1 generation, were observed in COPD patients [32].

Other FPR ligands, such as ANXA1 and SAA, are involved in cardiovascular disease progression. In fact, ANXA1 and its N-terminal peptides (Ac_2-26_ and Ac_9-25_) are endogenous anti-inflammatory and proresolving mediators, known to have significant effects in resolving inflammation in a variety of disease models and showing therapeutic potential in IRI [33]. ANXA1 has also protective role in myocardial infarction (MI) and stroke [34]. SAA is a sensitive marker of an acute inflammatory state [35] and although it is mainly associated with amyloidosis, it is involved in early atherogenic processes [36] and represents a marker of cardiovascular events [37,38].

Reactive oxygen species (ROS) are critical signaling molecules involved in both pathological and physiological cardiovascular processes. Several cytosolic sources contribute to the intracellular ROS pool, such as NADPH oxidase (NOX), xanthine oxidase, cyclooxygenases, and cytochrome P450 enzymes. Mitochondrial sources of ROS include the respiratory chain, monoamine oxidases, p66shc, and NOX4 [39]. Elevated levels of ROS result in oxidative stress and damage to DNA, lipids and proteins, as well as in impairing mitochondrial functions, thus contributing to cell death. Dysregulation of ROS generation and consequent oxidative stress has been observed in different cardiovascular diseases, including cardiac hypertrophy [40], heart failure [41], cardiac IRI [42], diabetic cardiomyopathy [43], and cardiomyopathy associated with Duchenne/Becker muscular dystrophy [44].

Since ROS play a crucial role in cardiovascular physiopathological processes, several studies are focused on identifying the molecular mechanisms of regulation of ROS production, as well as on developing therapies to counteract ROS production in heart diseases. In leukocytes and polymorphonucleate cells (PMNs) FPRs modulate oxidative burst through NOX-dependent ROS generation [45]. Indeed, FPRs stimulation triggers multiple phosphorylations of intracellular signaling molecules, such as extracellular signal-related kinases 1/2 (ERKs), protein kinase C (PKC), protein kinase B (PKB), mitogen-activated protein (MAP) kinases p38 (p38MAPK), phosphatidylinositol-3-kinase (PI3K), and phospholipase C (PLC), as well as nonsignaling proteins, such as p47phox and p67phox, required for NOX activation [45,46]. In other cell types, NOX-dependent ROS generation triggered by FPR1 and FPR2 stimulation is necessary to modulate several intracellular redox signaling pathways [47]. In fact, ROS can act as second messengers, by inhibiting protein tyrosine phosphatase (PTPs) activity and, in turn, promoting the phosphorylated state of protein tyrosine kinases (PTKs), thus regulating intracellular signaling cascades [48]. In support of these comments, a phospho-proteomic analysis on FPR2-stimulated cells identified 290 differentially phosphorylated proteins, including kinases and phosphatases, as new phosphorylation sites which, at least in part, are regulated by NOX-mediated ROS generation [14,45,46]. Furthermore, NOX-dependent transactivation of several Tyrosine Kinase Receptors (TKRs) by FPR1 or FPR2 stimulation [48] has been described in epithelial [49,50], neuronal [51] and endothelial cells [52].

Herein, we discuss the contribution of FPRs in physio-pathological processes associated with the progression of cardiovascular disease, describing detrimental and beneficial effects of the main FPRs agonists and their potential role as new therapeutic and diagnostic targets.

## 2. Formyl-Peptide Receptor 1

FPR1 shows the highest binding affinity for N-formylated peptides among all members of the FPRs family. Its stimulation results in PI3K, PKC, p38MAPK, and ERKs activation, as well as in increase of chemotaxis and NOX-dependent ROS production [53,54]. Both FPR1 and FPR2 cooperate with other GPCRs to modulate chemotaxis [10], as demonstrated by the observation that neutrophil accumulation in tissue injury is controlled by multiple chemoattractants, such as IL-8 (CXCL8), CXCL7 and CXCL1 [10]. However, FPR1 and FPR2 act as the first players in sensing chemotaxis signals resulting in rapid neutrophil infiltration [55]. In fact, their activation by the agonists produced at the injury site triggers a signaling cascade that culminates in the neutrophil migration, increased phagocytosis and superoxide release.

FPR1 is also expressed in several tumors. In highly malignant glioblastoma multiforme (GBM) cells it responds to the endogenous chemotactic ligand ANXA1 released by necrotic GBM cells [56]. In these cells, activated FPR1 enhances the survival, invasiveness, and production of angiogenic factors by cooperating with the epidermal growth factor receptor [56,57]. FPR1 is implicated in the production of angiogenic factors also in human liver cancer cells, where it promotes cell invasion and proliferation [58]. In human breast cancer cells, FPR1 and FPR2 interact with ANXA1 to enhance tumor cell proliferation [59]. Furthermore, in human gastric cancer (GC), FPR1 levels are correlated with more aggressive submucosal and serosal invasion accompanied by worse outcomes in patients [60]. Interestingly, FPR1 silencing significantly enhances GC tumorigenicity, suggesting that this receptor exerts a tumor suppressor function by inhibiting angiogenesis [57].

FPR1 contributes to endothelial/cardiac/cerebral dysfunction and repair in intermittent hypoxia/reoxygenation (IHR)/IRI [61,62,63], which is a common feature of several diseases, such as stroke and MI. Ischemia refers to a decrease in blood flow. Reperfusion injury is instead associated with initial blood-borne neutrophil infiltration, giving rise to an inflammatory response that results in tissue injury. In fact, the damaged tissue displays important signs of inflammation and microvascular injury that, unless resolved, lead to tissue dysfunction. Restoration of blood flow to a previously ischemic region is essential to prevent irreversible tissue damage but it is not always beneficial. During reperfusion, a great amount of damage occurs to the tissue, although a significant level of injury occurs due to ischemia itself. Several events are involved in the inflammatory cascade following myocardial injury, including leukocyte activation, lipid peroxidation and an increase of vascular permeability [64]. Leukocyte recruitment occurs in the microvasculature and involves a complex multistep cascade [63]. FPR1 gene deficiency reduces the risk of heart injury induced by IRI [65], as demonstrated by a significant decrease of collagen volume fraction, infarct area and apoptotic index observed in the heart of FPR1-silenced mice. This correlates with a reduction of p38MAPK, ERKs, jun kinase (JNK), MMP-2, TIMP-2, NF-kB, Bax, phospho-p38MAPK, phospho-ERKs, phospho-JNK levels, and cell apoptosis rate, as well as with an enhanced Bcl-2 level, cell proliferation and cell cycle progression. Therefore, these observations suggest that FPR1silencing reduces inflammation, cardiomyocyte apoptosis and ventricular remodeling in IRI, through the suppression of the MAPK signaling pathway [66]. Consistently, FPR1 expression levels are higher in patients with cardiac IRI [66].

Blockade of FPR1 functions in preventing IRI has been observed also in the liver. Mitochondrial DAMPs, including formyl peptides, are recognized by FPR1 [21] and the signaling triggered by this receptor is responsible for regulating neutrophil chemotaxis, which allows their migration into the necrotic area in hepatic ischemia-reperfusion [67]. Pretreatment with cyclosporine H (CSH), a selective FPR1 antagonist, prevents hepatic IRI as demonstrated by decreased serum transaminase and inflammatory cytokine levels, reduced hepatocyte necrosis/apoptosis and oxidative stress in a mouse model. Moreover, FPR1 blockade also inhibits the accumulation of neutrophils in the necrotic area [67].

IRI occurs in a sterile environment and involves acute inflammation and innate immune cell activation, leading to rapid infiltration of neutrophils. Therefore, inflammatory responses, that crucially contribute to reperfusion injury, are, at least in part, associated with circulating neutrophils activation. FPR1 plays an important role in neutrophil function and its expression is rapidly upregulated in response to inflammatory stimuli, which contribute to tissue damage through several mechanisms: (i) FPR1-mediated NOX-dependent ROS generation; (ii) release of elastases, cathepsin G, proteinase and other proteolytic enzymes; (iii) release of cytokines from local cells and subsequent neutrophil recruitment; (iv) obstruction of capillaries by neutrophils thus contributing to the no-flow phenomenon [68]. Therefore, FPR1 and the neutrophil-mediated inflammatory cascade during reperfusion represent an important target for therapeutic intervention.

Moreover, development of resistant to degradation FPR agonists as potential therapeutics could be useful for a range of inflammatory disorders. In particular, Cmpd17b is a small biased FPR1/FPR2 agonist that significantly reduces necrosis in cardiomyocytes subjected to hypoxia–reoxygenation [69].

ANXA1 is expressed constitutively in many cells, including neutrophils [68]. It is released upon neutrophil adhesion to endothelial cells, and it binds to FPRs receptors to suppress inflammatory signaling cascades [68]. Exogenous administration of ANXA1 exerts protective and anti-inflammatory actions and acts as a second messenger for many glucocorticoid effects in neutrophils, macrophages and other circulating inflammatory cells. Many N-terminal ANXA1-derived peptides, such as Ac_2-26_, display similar activity to the full-length protein, showing potent inhibition of neutrophil function and thus representing cardioprotective factors against myocardial IRI [68]. In particular, Ac_2-26_ preserves inotropic responsiveness at the level of ventricular muscle and contractile function of cardiac muscle in vitro [70,71,72,73]. The anti-inflammatory properties of ANXA1-derived peptides have been largely attributed to their anti-neutrophil actions in vivo and to activation of members of the FPR family [70]. ANXA1 affects myocardial function and is an endogenous regulator of post-ischemic recovery of left ventricular (LV) function. Ac_2-26_ rescues LV function on reperfusion, via activation of FPR1. Therefore, ANXA1-based therapies may represent a novel clinical approach for the prevention and treatment of myocardial reperfusion injury [74].

FPR1 is a potential biomarker for acute MI. In fact, it has been identified, together with genes involved in inflammation, as a differentially expressed gene in human MI blood tissues, compared with normal blood tissues [75,76]. In addition, FPR2 activation is involved in cardiac repair after MI. This suggests that the deregulated FPRs expression in blood tissue might account for the occurrence of acute MI [62,76]. Thus, FPRs represent novel therapeutic targets in MI, where inflammation is a major contributing mechanism. Cardiomyocyte survival and preservation of LV function seem to be enhanced by FPR1 [74], whereas FPR2 is responsible for attenuation of inflammation [77]. Therefore, dual FPR1/FPR2 agonists may be useful for lessening MI injury [69].

The spleen contributes to inflammatory responses during cardiac remodeling after MI, as demonstrated by the observation that the cardio-splenic axis plays a pivotal role in exacerbating MI size during post-ischemic reperfusion [78]. Interestingly, FPR1 blockade prevents the activation of this cardio-splenic signaling axis and abrogates the reperfusion-induced exacerbation of infarct size. In fact, myocardial neutrophil infiltration is reduced by cFLFLF, a specific FPR1 antagonist, which abrogates the infarct-exacerbating effect in mice. Consistently, in mice sham spleens the injection of ischemic heart homogenate significantly increases FPR1 expression [78].

Multiple genetic determinants and environmental factors contribute to modulate blood pressure (BP) levels [79]. Genome-wide analysis studies have identified candidate loci on the long arm of chromosome 19 (19q) harbors linked to increased BP levels and related pathological phenotypes [80,81]. FPR1 gene localizes in position 19q 13.3 and seems to play a key role in the physiopathology of hypertension, being crucially involved in the modulation of inflammation and in BP related metabolic pathways [82]. Polymorphisms of FPR1 are associated with inflammation and BP. In particular, FPR1 C32T (rs5030878), a nonsynonymous coding single nuclear polymorphism (SNP)(Ile11Thr), results associated with increased C-reactive protein (CRP) levels linearly related to BP [83,84,85].

Abdominal aortic aneurysm (AAA) is characterized by a progressive aortic dilatation and weakening of the vascular wall that may provoke an aortic rupture, which often is fatal. Pathogenesis of AAA includes extracellular matrix degradation, vascular smooth muscle cell de-differentiation/apoptosis, ROS accumulation, and inflammatory cell infiltration [86,87]. PMNs infiltration and activation, and chemokine-mediated neutrophil recruitment contribute to the pathogenesis of AAA and may serve as diagnostic and therapeutic targets [88,89,90]. For instance, the chemokine-like factor family with sequence similarity 3, member D (FAM3D), a dual FPR1/FPR2 agonist, is markedly upregulated in human AAA tissues [90], and cinnamoyl-F-(D)L-F-(D)L-F-K (cFLFLF), a PEGylated peptide ligand that binds FPR1 on activated neutrophils, allows the early, accurate and noninvasive diagnosis of AAA [91].

Platelets are the main cells which regulate hemostasis and thrombosis. They play crucial roles in mediating MI, stroke, and venous thromboembolism (VTE). Platelets are also involved in the “immunothrombosis”, a coordinated intravascular coagulation response in which platelets and immune cells prevent dissemination of pathogens leading to activation of the innate and adaptive immune response [92]. Consistently, several viral or bacterial infections contribute to the risk of thrombosis that manifests as arterial thrombosis or VTE. Therefore, although immunothrombosis is an efficient way of assisting the immune system, it may significantly contribute to the overall risk of cardiovascular disease [92]. FPR1 is also expressed on the platelets membrane, and the binding of N-formyl peptides triggers their chemotactic and migratory response [93]. For instance, N-fMLF primes platelet activation increasing the risk of thrombus formation. *Fpr1*-deficiency in mice or inhibition using a pharmacological inhibitor in human platelets results in diminished agonist-induced platelet activation [94]. This suggests that FPR1 influences the modulation of platelet activation and thrombus formation and could represent a new molecular target able to control platelet-mediated complications in several pathological settings.

ANXA1, that binds both FPR1 and FPR2, plays a modulatory role in platelets. In fact, ANXA1 administration significantly decreases both platelet adherence to the inflamed cerebral endothelium after stroke and regulates the state of platelet activation. ANXA1 also reduces platelet–platelet aggregate formation, decreases thromboxane B2 production and phosphatidylserine expression and, thus, the prothrombotic potential of platelets. It also reduces the propensity for platelets to aggregate and causes thrombosis by affecting integrin αIIbβ3 levels, thereby reducing the risk of thrombotic events. These observations show a multifaceted role for ANXA1 to act both as a therapeutic and a prophylactic drug via its ability to promote endogenous proresolving, anti-thromboinflammatory circuits in cerebral IRI [95].

**Figure 1:**
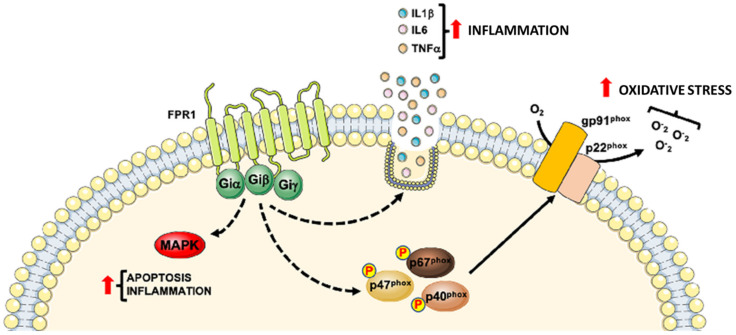
Detrimental role of Formyl Peptide Receptor 1 (FPR1) in CVDs progression. FPR1 contributes to CVDs progression by promoting the inflammatory state through the activation of MAPKs pathway, generation of reactive oxygen species and the release of pro-inflammatory cytokines.

Table 1 summarizes the role of FPR1 and Figure 1 shows the intracellular signaling pathways mainly involved in cardiovascular diseases (CVDs).

## 3. Formyl-Peptide Receptor 2

FPR2 is the most promiscuous member of the FPRs family for its ability to recognize a broad variety of ligands of different origins, including proteins, peptides and lipids [45]. Intracellular signaling cascades mediated by FPR2 have been widely analyzed [96]. A phosphoproteomic analysis showed that FPR2 stimulation with proresolving agonists induces phosphorylation of several proteins involved in the regulation of cell cycle, cell division, apoptosis, and transmembrane transport [14,45,46]. FPR2 exerts proinflammatory effects in response to specific ligands, such as SAA or LL-37 [47], whereas mediates anti-inflammatory responses following Resolvin D1 (RvD1), LXA4, or ANXA1 binding [96]. The proinflammatory responses include superoxide production in neutrophils, chemotaxis of monocytes and neutrophils, chemokine CCL2 production in endothelial cells (ECs) and monocytes, and increase of CXCL8 expression in neutrophils [97,98].

Atherosclerosis is a chronic inflammatory process mainly occurring in the arteries’ intimal layer, particularly at bifurcation points of the blood vessel. The earliest events of atherosclerosis lesions include activation of the endothelium by different risk factors, including hypercholesterolemia. The events responsible for the clinical symptoms include the plaque formation, as a result of oxidized lipids creating foam cells. Following the mechanical injury to the endothelium, circulating leukocytes, monocytes, and T-lymphocytes attach and infiltrate the intima. The development of neointimal hyperplasia and restenosis is due to the release of mediators that promote smooth muscle cell migration towards lumen with fibrous cap formation [99]. Clinical manifestation of systemic atherosclerosis is associated with coronary artery disease, which is the leading cause of death in Western countries [100].

Much evidence suggests that FPR2 plays a pathogenic role in atherogenesis. For instance: (i) it is expressed in macrophages in human atherosclerotic lesions, where it accelerates atherosclerosis development through effects on bone marrow-derived cells [101]; (ii) FPR2 expression in the carotid artery is associated with clinical signs of cerebral ischemia and a more stable atherosclerosis plaque phenotype [101]; (iii) FPR2 expression in smooth muscle cells (SMC) is coupled to increased collagen production and maturation, and to pathways of decreased collagen degradation [101]. Furthermore, in both low-density lipoprotein receptor null (LDLR^−/−^) and apolipoprotein E deficient (ApoE^−/−^) mice, genetic disruption of FPR2 results in reduced atherosclerosis development [101,102]. Similarly, atherogenic FPR2^−/−^ mice develop fewer lipid streaks in the descending aorta [101]. Taken together, these observations suggest that FPR2 signaling has important vascular effects and contributes both to increased atherosclerotic lesion and a more stable plaque phenotype.

However, in different hyperlipidemic murine models, genetic targeting of FPR2 has generated conflicting results. In fact, in ApoE and FPR2 double-knock-out mice increased lesions size in early stages of atherosclerosis have been described [103]. Furthermore, ANXA1 treatment of LDLR^−/−^ mice reduces atherosclerotic plaque burden [104], and Ac_2-26_ prevents chemokine-mediated integrin activation in neutrophils and monocytes, resulting in a reduction in early atherogenesis and plaque formation [103].

The possible rationale for these differences may be the use of proinflammatory or proresolving FPR2 agonists in the different experimental models [105]. In fact, in LDLR^−/−^ mice the levels of the proinflammatory FPR2 ligand SAA, which is a well-known biomarker of cardiovascular risk, are approximately 10,000-fold higher compared with LXA4, an anti-inflammatory FPR2 agonist. This suggests that potential inflammation resolution induced by LXA4 signaling through FPR2 may be impaired in this atherosclerosis model, as result of the low biosynthesis of this agonist [101]. Overall, those findings highlight a failure in the resolution of inflammation in atherosclerosis, evidenced by decreased levels of proresolving ligands and increased levels of proinflammatory FPR2 agonists. Accordingly, ANXA1 fragment Ac_2-26_ administration in vivo significantly reduces atherosclerotic lesion sizes and macrophage accumulation in an FPR2 dependent manner [103], and the delivery of nanoparticles containing the proresolving ANXA1 mimicking peptide Ac_2-26_ reduces experimental atherosclerosis in presence of a functional FPR2 [106]. Similarly, intraperitoneal administration of human recombinant ANXA1 in LDLR^−/−^ mice significantly attenuates the progression of existing atherosclerotic plaques through FPR2-dependent reduction of neutrophil rolling and adhesion to activated endothelial cells [104]. Furthermore, studies performed in Fpr1-null mouse strain have revealed that FPR1 does not mediate the cardioprotection provided by Ac_2-26_ peptide [65]. These observations highlight the role of FPR2 in the signal transduction cascade responsible for the resolution of inflammation in response to appropriate ligand stimulation and indicate that stimulating proresolving signaling through FPR2 may be a therapeutic option for atherosclerosis [107].

Other proresolving or anti-inflammatory FPR2 agonists are therapeutic in atherosclerosis. In fact, in ApoE^−/−^ mice, LXA4 treatment blocks atherosclerosis progression in the aortic root and thoracic aorta. In these mice, FPR2 mediates the reduction of macrophage infiltration and of apoptotic cells in atherosclerotic lesions [102]. WKYMVm, a synthetic anti-inflammatory FPR2 agonist, can recruit endothelial progenitor cells contributing to neovascularization [108,109] and promoting re-endothelializations, as well as inhibiting restenosis [110], thus showing therapeutic activities [111].

On the other hand, in human atherosclerotic lesions, SAA elicits a proinflammatory/procoagulation response, as well as the release of proinflammatory cytokines, chemokines and adhesion molecules [112]. FPR2 is upregulated in blood mononuclear cells from atherosclerosis patients and SAA stimulation induces macrophage foam cell formation, JNK activation and lectin-like oxidized low-density lipoprotein receptor 1 upregulation [113,114]. In vascular endothelial cells, enhanced aggregation of oxidized low-density lipoprotein (ox-LDL) is one of the major changes in atherosclerotic lesions. In these cells, FPR2 promotes autophagic degradation of oxLDL by interacting with humanin and inducing LCR3-II protein expression and reducing p62 protein level [115].

FPR2-SAA interaction contributes to atherosclerosis progression also by promoting the secretion of long pentraxin 3 (PTX3), an essential component of innate immunity, in human aortic endothelial cells [116]. Interestingly, the proinflammatory peptide LL-37, which efficiently binds FPR2 [47], is also upregulated in human atherosclerotic lesions [117], but its role remains to be further investigated. FPR2 stimulation by LL-37 also contributes to prime circulating platelet and induces thrombo-inflammation [118,119].

ANXA1 and its N-terminal peptides are protective in several models of IRI [33]. In fact, infusion of recombinant ANXA1 decreases myocardial infarct size [120] and the treatment with Ac_2-26_ reduces both infarct size, myeloperoxidase (MPO) and IL-1β levels [121]. Intra-cerebroventricular infusion of Ac_2-26_ decreases stroke volume and cerebral edema in rats [122], by reducing leukocyte–endothelial interactions [123]. In mice, Ac_2-26_ also attenuates neutrophil and platelet activation and neutrophil–platelet aggregation in cerebral microvasculature after induction of cerebral IRI [124]. The ability of Ac_2-26_ to preserve cardiomyocyte contractility is related to the activation of PKC, p38MAPK, and ATP-sensitive potassium (KATP) channels [70].

In IRI, FPR2 promotes the resolution of inflammation also by interacting with the proresolving lipid mediators RvD1. This resolvin is generated in response to vascular injury in primary endothelial cells and vascular smooth muscle cells, eliciting anti-inflammatory autocrine and/or paracrine signaling, at least in part through FPR2 [125]. In fact, RvD1 administration, prior to ischemia-reperfusion, significantly inhibits inflammatory cascades, as observed by reduced IL-6, TNF-α and MPO levels. Moreover, RvD1 administration also reduces apoptosis, through Akt phosphorylation [126], and attenuates IR-induced damage. These effects depend on FPR2 interaction since they are abolished by FPR2 silencing or pretreatment with FPR2 antagonists [127]. In an animal model of hypoxia-ischemia, FPR2 activation with RvD1 triggers Rac1/NOX2 signaling pathway and significantly reduces percent infarction area ameliorating short- and long-term neurological deficits [128].

Leukocyte–endothelial (L-E) interactions is a crucial event in IRI contributing to modulate inflammatory response [129]. The anti-inflammatory mechanism exerted by FPR2 is also reflected in the reduction of L-E interactions after treatment with Ac_2-26_ or 15-epimer-LXA4, that is prevented by pretreatment with Boc2, an FPR1/FPR2 antagonist, or WRW4, an FPR2 antagonist [123,124]. These studies provide further evidence of a beneficial effect of the FPR2 pathway in inflammation.

Since FPR2 is considered a putative target for IRI [63], a number of novel ligands showing protective effects have been identified. These include the FPR2 agonist CGEN-855A, which shows cardioprotective effects in rat and murine myocardial IRI, similar to those reported for the ANXA1 mimetic peptide Ac_2-26_ [130]. Furthermore, 15-epi-16-(*p*-fluorophenoxy)-LXA4-methyl ester [15-epi-16-(FPhO)-LXA4-Me] is an FPR2 agonist that exhibits protective effects in renal IRI models, by modulating cytokine and chemokine expression and neutrophil recruitment [131]. Cmpd17b is a biased FPR1/2 agonist that reduces inflammatory responses associated with reperfusion after an acute MI [69]. Finally, the novel 3-oxa-aspirin-triggered 15-epi-lipoxin analogues, ZK-994 and ZK-142, show inhibition of neutrophil accumulation in murine hind-limb IRI-induced second-organ lung injury by binding FPR2 [132]. Table 2 summarizes FPRs ligands developed as therapeutic drugs in cardiovascular diseases.

FPR2 protective effects are mainly focused on neutrophil responses, and neutrophils chemoattractants receptors, such as FPRs, also play a key role in AAA expansion [124,133]. In fact, ANXA1, LXA4, and RvD1 levels are increased in patients undergoing surgical AAA repair [134], and D-series resolvins inhibit aortic dilatation in experimental murine models of AAA [135]. On the other hand, FPR2 deficiency or disrupting lipoxin and resolvin formation by genetic deletion enhances AAA and increases aneurysmal leukocyte infiltration [136], thus reinforcing the protective role of lipoxin formation and FPR2 signaling in AAA.

Unresolved inflammation can contribute to the development of heart failure following MI and anti-inflammatory FPR2 ligands show beneficial effects. In fact, in a murine model of acute MI, administration of the synthetic FPR2 agonist WKYMVm provides cardiac protection by mobilizing circulating angiogenic cells, such as endothelial progenitor cells, thus contributing to their homing to ischemic heart in an FPR2-dependent manner [62]. Post-MI healing occurs in two phases: the early acute inflammatory phase and the resolving phase. The early activation of the resolving phase is essential to resolve the inflamed infarcted area post-MI [137]. Interestingly, FPR2 interaction with 15-epimer-LXA4 shows protective functions in MI treatment by triggering early activation of the resolving phase, thereby improving left ventricular function post-MI [138]. Accordingly, FPR2 inactivation by WRW4 leads leukocytes to nonresolving inflammation in acute MI [139].

FPR2 has a significant impact on the survival, structure, and function of cardiometabolic and renal syndromes. Furthermore, it serves as a key resolution sensor for heart function, renal homeostasis and lipid metabolism in cardiovascular physiology. In fact, lack of FPR2 leads to the development of spontaneous obesity and diastolic dysfunction with reduced survival with aging. After a cardiac injury, FPR2^−/−^ mice show a lower expression of lipoxygenases and a reduction in RvD1 in the infarcted left ventricle, indicating nonresolving inflammation. At the cellular level, FPR2^−/−^ mice have impairment of macrophage phagocytic function ex-vivo with an expansion of neutrophils after myocardial infarction. This suggests that blockade of FPR2 functions impairs biosynthesis of specialized proresolving mediators amplifying unresolved inflammation after cardiac injury [140].

Therefore, the biology of FPR2 has stimulated several medicinal chemistry programmes and FPR2 agonists have been also tested in humans [77,141]. In fact, synthetic lipoxin mimetics have been investigated in preclinical settings [142] and a phase I trial has been conducted with ACT-389949, a small-molecule FPR2 agonist [143]. The most reasonable application of FPR2 agonists is addressed to heart failure rather than classic inflammatory pathologies. Indeed, a Phase I trial has been concluded for the compound BMS-986235, a selective FPR2 agonist with tissue-protective properties in experimental heart failure [144]. ANXA1 and its peptides are endogenous anti-inflammatory mediators of glucocorticoids that show good efficacy in many diseases associated with acute or chronic inflammation. ANXA1 protects peripheral organs against the injury and dysfunction caused by hyperglycemia or hyperlipidemia. Nevertheless, translational studies in humans have yet to be performed. However, in preclinical settings, ANXA1 shows a direct action on cardiac macrophage polarization in the post-infarct heart, through induction of a proangiogenic macrophage phenotype [145]. The first-generation of FPR2 small-molecule agonists also show heart-protective properties in acute myocardial infarct [69] and compound 43, a dual FPR1/FPR2 agonist, provides a high degree of protection in a model of heart failure [146]. The beneficial responses to FPR2 activation in infarction have been linked both to tissue-mediated effects, such as focused on myocardial macrophages and to systemic responses, such as the ability to mobilize angiogenic cells [3,62] (Table 2).

**Table 2:** FPRs ligands developed as therapeutic drugs in cardiovascular diseases (CVDs).

Compound	Interaction	Therapeutic Effects
Cmpd17b	Small biased FPR1/FPR2 agonists	Reduction of necrosis in cardiomyocytes subjected to hypoxia–reoxygenation exerting a cardioprotective effect [69]
		Reduction of inflammatory responses associated with reperfusion after an acute MI [69]
ZK-994 and ZK-142	FPR2	Inhibition of neutrophil accumulation in murine hind-limb IRI-induced second-organ lung injury [132]
ACT-389949	FPR2	Protection against heart failure [143]
CGEN-855A	FPR2	Cardioprotective effects in rat and murine myocardial IRI, similar to those reported for the ANXA1 mimetic peptide Ac_2-26_ [130]
BMS-986235	FPR2	Protective properties in experimental heart failure [144]
compound 43	Dual FPR1/FPR2 agonist	High degree of protection in a model of heart failure [146]
PLGA microspheres encapsulating WKYMVm	FPR2	Induction of neovascularization in vivo hind limb ischemia model [109]

**Figure 2:**
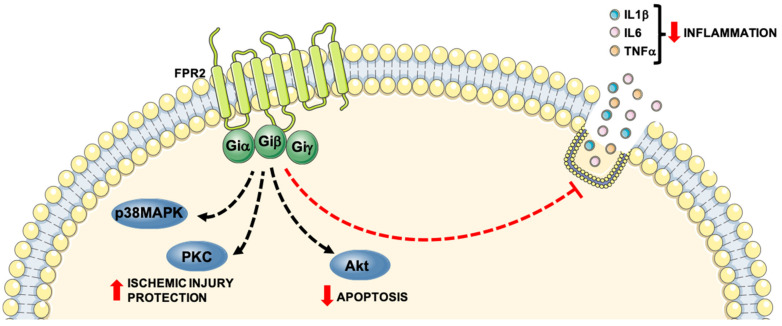
Protective role of Formyl Peptide Receptor 2 (FPR2) in CVDs progression. FPR2 stimulation with proresolving ligands exerts a protective role in CVDs progression by inhibiting release of proinflammatory mediators and preserving cell functions from ischemic injury mainly due the activation of p38MAPK, PKC and Akt.

These studies support the evidence that proresolving mediators exert beneficial effects through FPR2, with implications for several cardiovascular diseases. For instance, the failure in the resolution of inflammation mediated by limited production of proresolving lipid FPR2 ligands, as well as the unmasking of proinflammatory FPR2 signaling, may be responsible for chronic inflammatory reactions in atherosclerosis. Restoring the proresolving FPR2 signaling may offer novel therapeutic options both in the prevention of atherosclerosis progression and in increasing atherosclerotic plaque stability. In addition, improving FPR2 signaling could be considered in the context of limiting neutrophil-mediated IRI. Table 3 summarizes the role of FPR2 and Figure 2 shows the intracellular signaling cascades mainly involved in cardiovascular diseases.

## 4. Formyl-Peptide Receptor 3

FPR3 is expressed in eosinophils, monocytes, macrophages, and other cells and is the less known isoform of the FPR family. Its functional role is still unclear, and its ligands are not completely identified. FPR3 is unresponsive to formyl peptides, but shares a low affinity for several FPR2 agonists including ANXA1 fragment Ac_1-25_, *Helicobacter pylori*-derived peptide Hp_2-20_ and the synthetic peptide WKYMVm [147]. Therefore, FPR3 may have its own unique functional significance. Among identified ligands, an acetylated N-terminal fragment of the human heme-binding protein, named F2L [147], and the neuroprotective peptide humanin are the better characterized FPR3 agonists. FPR3 is mainly localized in small intracellular vesicles and is highly phosphorylated, a signal that indicates receptor inactivation and internalization [148]. This suggests that FPR3 rapidly internalizes after ligands’ binding and thereby may serve as a “decoy” receptor to reduce the binding of its ligands to other receptors [53,148].

## 5. Conclusions

Pathological vascular conditions, arising from prolonged atherosclerosis, coronary artery disease, diabetes, uncontrolled hypertension, and aging cause many acute cardiovascular events. Behavioral and psychosocial factors can also trigger and contribute to ischemic stroke and MI, which require rapid restoration of blood flow followed by subsequent anti-inflammatory strategies and cardio-protection for secondary prevention. However, recovery of blood flow and reoxygenation lead to a further increase of tissue injuries. To restore homeostasis, the resolution of inflammatory and thrombotic environment is critical and molecules with proresolving properties have been proposed as anti-inflammatory and proresolving mediators. Many of these bind members of the FPR family, which belong to the classic chemoattractant GPCRs and play a crucial role in inflammation. FPRs have multiple and different ligands and they are expressed by a great variety of cell types. These ligands may originate from pathogens, hosts, synthetic peptides, or compound libraries, or even nonhost multicellular organisms. Since FPRs show diverse agonist binding capacity, it is not surprising that they may be either detrimental or beneficial in different pathophysiological conditions. In inflammatory states, FPRs participate in chemotaxis, degranulation, ROS production, promoting neutrophil–platelet interactions and allowing apoptosis and phagocytosis. FPR1 signaling is associated with oxidative burst and mitochondrial-derived FPR1 ligands act as chemotactic DAMPs, activating the innate immune system. FPR2 is the most versatile member of the FPR family and can interact with a multitude of ligands resulting in both anti-inflammatory, proresolving and proinflammatory functions. In addition to the high number of agonists, there are also extensive lists of antagonistic ligands that may also provide protective mechanisms in several diseases. These findings greatly highlight the role of FPR biology and future lines of investigation on FRPs in cardiovascular diseases should be focused on autophagy mechanisms associated with IRI, cardiac cell metabolism triggered by FPRs, as well as in proangiogenetic molecular mechanisms. Further studies examining the regulation, signaling, structural basis for ligand recognition, and on participation in pathophysiologic processes of FPRs, should be conducted to provide new insights into their potential as therapeutic targets for diseases.

## Figures and Tables

**Table 1:** FPR1 involvement in cardiovascular diseases (CVDs).

CVDs	Expression and/or Stimulation	Role
Ischemia reperfusion injury (IRI)	FPR1 deficiency ^m^	Protective effects by reducing the risk of heart injury induced by IRI [65]
	FPR1 silencing ^r^	Protective effects mediated by depression of inflammation, cardiomyocyte apoptosis and ventricular remodeling in rats with I/R injury through the suppression of the MAPK signaling pathway activation [66]
	FPR1 antagonist CSH ^m^	Protective effects by reducing hepatocyte necrosis/apoptosis, and diminishing inflammatory cytokine, chemokine, and oxidative stress levels as well as accumulation of neutrophils in the necrotic area [67]
	FPR1 stimulation with ANXA1 ^m and r^	Cardioprotective role by preserving inotropic responsiveness at the level of ventricular muscle and contractile function of cardiac muscle in vitro [70,71,72,73]
	FPR1 blockade ^m^	Beneficial effects mediated by abrogation of reperfusion-induced exacerbation of infarct size [78]
Blood pressure (BP) levels	*FPR1* C32T (rs5030878) single nuclear polymorphism (SNP) ^h^	Negative prognostic factor and detrimental effects associated with increased C-reactive protein (CRP) levels linearly related with BP [83,84,85]
Abdominal aortic aneurysm (AAA)	FPR1 involvement ^m^	Detrimental effects since FPR1 results involved in neutrophil recruitment and aggravated AAA development [91]
Acute myocardial infarction (AMI)	*FPR1* as a differentially expressed gene (DEG) ^h^	Role has to be determined even if FPR1 results a DEG in human AMI blood tissue, compared with normal blood tissue using microarray data [75]
		Potential beneficial role for AMI prevention [76]
Platelet-mediated complications	FPR1 inhibition or gene deletion ^m and h^	Detrimental effects by impairing agonist-induced platelet activation in *Fpr1*-deficient mice or in pharmacologically FPR1 inhibited human platelets [94]
Endothelial cell function and HUVECs	FPR1 stimulation with NfMLF ^hc^	Beneficial effects by promoting proliferation and capillary network formation
Angiogenesis	FPR1 stimulation with ANXA1 ^hc^	Beneficial effects by inducing angiogenesis and/or production of angiogenic factors [56,57,58]

Table legend: ^m^ = mouse; ^r^ = rat; ^h^ = human; ^hc^ = human cell.

**Table 3:** FPR2 involvement in cardiovascular diseases (CVDs).

CVDs	Expression and/or Stimulation	Effects
Atherosclerotic lesions	FPR2 stimulation with SAA ^hc and mc^	Detrimental effects contributing to atherosclerosis progression in human aortic endothelial cells (HAECs) [116]
		Detrimental effects contributing to atherosclerosis progression upregulating the secretion of long pentraxin 3 (PTX3) in human aortic endothelial cells [116]
		Detrimental effects by upregulating oxidized low-density lipoprotein (oxLDL) contributing to macrophages differentiation into foam cells and in turn inflammatory cytokine production and plaque formation [114]
	FPR2 mRNA levels ^h and m^ (up-regulated expression)	Dual role by promoting both disease progression (detrimental) and plaque stability (beneficial) [101]
	FPR2 stimulation with AnxA1 ^m^	Protective role by reducing sizes and macrophage accumulation in the atherosclerotic lesion [103]
		Protective effects by reducing the progression of existing plaques of aortic arch and subclavian artery by FPR2 dependent reduction of neutrophil rolling and adhesion to activated endothelial cells [104]
		Protective effects exerted by proresolving ANXA1 mimicking peptide Ac_2-26_ reduces experimental atherosclerosis in presence of a functional FPR2 [106]
	FPR2 stimulation with lipoxinA4 ^m^	Protective effects by reducing macrophage infiltration and apoptotic cells in atherosclerotic lesions [102]
	FPR2 stimulation with LL-37 ^m^	Detrimental effects by contributing to plaque formation by priming circulating platelet and inducing thromboinflammation [118,119]
Neovascularization	FPR2 stimulation with WKYMVm ^hc, m and rb^	Beneficial effects by recruiting endothelial progenitor cells, contributing to neovascularization and promoting re-endothelialization [108,109]
		Protective effects by inhibiting restenosis [110]
Ischemia reperfusion injury (IRI)	FPR2 stimulation with LXA4 or AnxaA1 ^m^	Protective effects by counter regulating the inflammatory response during IRI [123,124]
	FPR2 antagonist Boc2 ^m^	Detrimental effects exerted by pre-ischemia Boc2 administration resulting in LXA4 abrogated production and impaired vascular reactivity [124]
	FPR2 stimulation with Ac_2-26_^r^	Protective effects by preserving cardiomyocyte contractility related to the activation of PKC, p38, and KATP channels [70]
	FPR2 stimulation with SAA ^hc^	Detrimental effects, contributing to atherosclerosis progression by upregulating the secretion of long pentraxin 3 (PTX3) in human aortic endothelial cells [116]
	FPR2 stimulation with RvD1 ^r^	Protective effects by inhibiting inflammatory cascades; reducing IL-6, TNF-α and MPO levels; diminishing apoptosis by Akt phosphorylation, and attenuating IR-induced damage [126]
		Protective effects by reducing percent of infarction area, ameliorating short- and long-term neurological deficits trough the activation of Rac1/NOX2 signaling pathway [128]
	FPR2 stimulation with CGEN-855A ^r and m^	Cardioprotective effects in rat and murine myocardial IRI [130]
	FPR2 stimulation with 15-epi-16-(*p*-fluorophenoxy)-LXA4-methyl ester ^m^	Protective effects in renal IRI models, by modulating cytokine and chemokine expression and neutrophil recruitment [131]
	FPR2 stimulation with compound 17b ^m and mc^	Protective effects by reducing inflammatory responses associated with reperfusion after an acute MI [69]
	FPR2 stimulation with ZK-994 and ZK-142 ^m^	Protective effects by inhibition of neutrophil accumulation in murine hind-limb IRI-induced second-organ lung injury [132]
Abdominal aortic aneurysm (AAA)	FPR2 stimulation with LXA4 ^m^	Protective role by limiting neutrophil inflammation [135,136]
Myocardial infarction (MI)	FPR2 stimulation with WKYMVm ^m^	Cardiac protection by mobilizing circulating angiogenic cells, contributing to their homing to ischemic heart [62]
	FPR2 stimulation with 15-epimer-LXA4 ^m^	Protective effects by triggering early activation of the resolving phase and improving left ventricular function post-MI [138]
	FPR2 inactivation by WRW4 ^m^	Detrimental effects leading leukocytes to nonresolving inflammation in acute MI [139]
	Fpr2 gene deletion ^m^	Detrimental effects by impairing biosynthesis of specialized proresolving mediators amplifying unresolved inflammation after cardiac injury [140]

Table legend: ^m^ = mouse; ^r^ = rat; ^h^ = human; ^hc^ = human cell; ^mc^ = murine cell; ^rb^ = rabbit.

## Data Availability

The data presented in this study are available in the References herein reported.
